# Model-Based Optimization of Solid-Supported Micro-Hotplates for Microfluidic Cryofixation

**DOI:** 10.3390/mi15091069

**Published:** 2024-08-24

**Authors:** Daniel B. Thiem, Greta Szabo, Thomas P. Burg

**Affiliations:** 1 Integrated Micro-Nano-Systems Laboratory, Technische Universität Darmstadt, 64283 Darmstadt, Germany; greta.szabo@tu-darmstadt.de; 2 Centre for Synthetic Biology, Technische Universität Darmstadt, 64289 Darmstadt, Germany

**Keywords:** cryofixation, vitrification, cooling rate, heat conduction model

## Abstract

Cryofixation by ultra-rapid freezing is widely regarded as the gold standard for preserving cell structure without artefacts for electron microscopy. However, conventional cryofixation technologies are not compatible with live imaging, making it difficult to capture dynamic cellular processes at a precise time. To overcome this limitation, we recently introduced a new technology, called microfluidic cryofixation. The principle is based on micro-hotplates counter-cooled with liquid nitrogen. While the power is on, the sample inside a foil-embedded microchannel on top of the micro-hotplate is kept warm. When the heater is turned off, the thermal energy is drained rapidly and the sample freezes. While this principle has been demonstrated experimentally with small samples (<0.5 mm^2^), there is an important trade-off between the attainable cooling rate, sample size, and heater power. Here, we elucidate these connections by theoretical modeling and by measurements. Our findings show that cooling rates of 10^6^ K s^−1^, which are required for the vitrification of pure water, can theoretically be attained in samples up to ∼1 mm wide and 5 μm thick by using diamond substrates. If a heat sink made of silicon or copper is used, the maximum thickness for the same cooling rate is reduced to ∼3 μm. Importantly, cooling rates of 10^4^ K s^−1^ to 10^5^ K s^−1^ can theoretically be attained for samples of arbitrary area. Such rates are sufficient for many real biological samples due to the natural cryoprotective effect of the cytosol. Thus, we expect that the vitrification of millimeter-scale specimens with thicknesses in the 10 μm range should be possible using micro-hotplate-based microfluidic cryofixation technology.

## 1. Introduction

Micro-hotplates are widely used in the field of conductometric gas sensors [1,2,3]. These devices heat a metal oxide layer to specific temperatures, stimulating gas–surface interactions and changing the conductance, which is then measured to detect different gases. These hotplate designs are usually optimized for low power consumption [4,5,6,7]. This is achieved through a high isolation by suspending the hotplate with support beams, minimizing thermal contact with the substrate. Due to their low thermal mass, the transient response of these devices is typically very fast (up to <200 μs) [8,9,10,11,12,13]. Recent advances have further reduced the thermal mass by using nanowires to decrease power consumption while increasing the sensitivity of these gas sensors [14,15]. This power reduction is stated as a goal to improve the portability of such sensors [14]. While micro-hotplates for gas sensors can achieve fast transient responses [16,17] by optimizing the thermal isolation of the micro-heater; microfluidic heaters, in contrast, must heat the thermal mass of the sample itself, which inhibits a fast thermal response.

**Figure 1:**
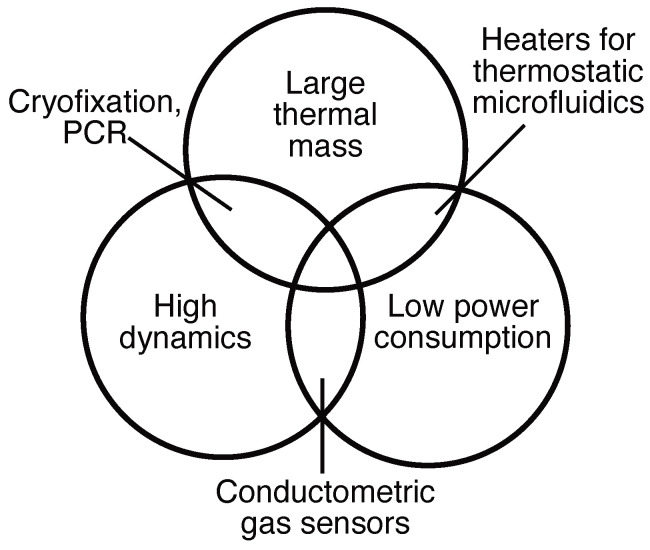
While conductometric gas sensors with micro-hotplates are commonly optimized for low power consumption and high dynamics, their thermal mass can be small. Cryofixation systems, on the other hand, aim to have a high dynamic behavior. Their thermal mass scales with the size of the sample. For cryofixation systems, the power consumption is not a strategic property.

Fast temperature changes of a thermal mass are used in a variety of microfluidic and microbiological applications. For example, rapid heating and cooling are needed to improve cycle time for polymerase chain reactions (PCRs) [18,19,20,21,22,23], droplet actuation using thermocapillary effects [24], or microfluidic actuation with microbubbles [25,26] or thermally expandable polymers [27]. The use of rapid temperature changes for the operation of thermal micromixers in microfluidic systems has also been proposed in the literature [28]. As illustrated in Figure 1, different applications require different trade-offs between the size, power consumption, and response time or rate.

In this work, we focus on high cooling rates of defined thermal masses on micro-hotplates, which are important for preparing biological samples using cryofixation in the field of structural biology. Here, the sample is rapidly cooled down to preserve its ultrastructure in amorphous, or vitreous, ice. This is enabled by the high cooling rates in cryofixation systems, which leave no time for ice crystals to form [29].Cryofixed samples can then be used for investigations using electron microscopy (EM) after freeze substitution, cryogenic electron microscopy (cryo-EM), and X-ray microscopy at cryogenic temperature. It is widely accepted that cooling rates (CRs) over 10^6^ K s^−1^ are necessary to enable the full vitrification of pure water. However, for cells and tissues, this requirement is somewhat relaxed depending on the degree of natural cryoprotection imparted by the high concentration of dissolved biomolecules [30,31]. Often, cooling rates of around 10^4^ K s^−1^ still allow the vitrification of the sample, sometimes with the formation of locally confined ice crystals of sufficiently small sizes to not perturb the structures of interest [32].

Over the past decades, various methods have been developed to enable the cryofixation of samples of different sizes. Plunge freezing is the most common method for the cryofixation of samples on transmission electron microscopy (TEM) grids [33]. The samples are blotted and plunged into a cryogen with a high cooling efficiency. The thickness of the water layer on top of the grids after blotting is usually less than 1 μm [34]. For samples on the order of 10 μm thick, slam freezing allows vitrification by quickly establishing thermal contact between the sample and a cold block [35]. Samples that are even thicker can only be vitrified using high-pressure freezing at 2000 bar [36].

**Figure 2:**
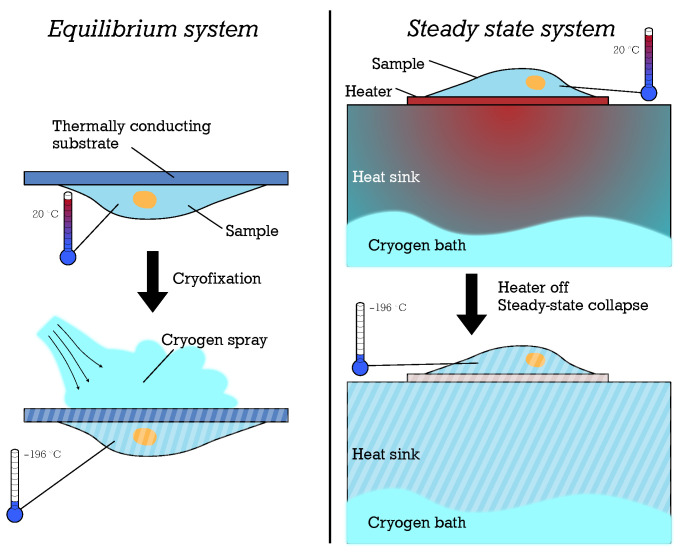
Two different principles for cryofixation with optical access have emerged. In an equilibrium system (**left**), the sample and the system are in a thermal equilibrium at room temperature until the cryofixation is started by spraying a cryogen on the substrate which rapidly cools the sample. A steady-state system (**right**) holds the sample at room temperature while a cryogen cools the backside of the system. This steady state collapses as soon as the heater is turned off and the sample is cryofixed.

Although plunge freezing and slam freezing provide reliable and consistent cryofixation, they do not allow optical access to the sample at the precise time of cryofixation due to the limitations of the working principle. Live imaging during cryofixation is often desirable for studying biological processes with sub-second dynamics, such as intraflagellar transport and neuronal firing [37]. In recent years, two design principles for cryofixation systems with optical access have emerged. In the first principle, which we refer to as *equilibrium systems*, the environment is initially warm and the sample is in thermal equilibrium with the environment. The sample is mounted to an optical microscope on a thermally well-conducting substrate in thermal equilibrium with the warm environment (see Figure 2 left). Freezing is accomplished by spraying a cryogenic gas or liquid jet (e.g., liquid nitrogen or cold Helium gas) on the backside of the sample holder and then immersing the sample in liquid nitrogen [38]. In the second principle, which we refer to as *steady-state systems*, the sample is initially maintained far from thermal equilibrium through a micro-hotplate that is counter-cooled with liquid nitrogen (see Figure 2 right) [39]. When the micro-hotplate is turned off, the heat in the sample is quickly absorbed by the cold heat sink below.

This paper proposes a modeling approach for thermal conduction in a steady-state system. We also examine the possibility of increasing the size limits of this system while maintaining cooling rates >10^6^ K s^−1^, as well as the maximum thickness of aqueous samples for cryofixation with cooling rates >10^4^ K s^−1^. For this investigation, the cooling rate is defined as the inverse of the time between the start of the cooling process (20 °C) and the time when the sample reaches a critical temperature of −90 °C.

## 2. Fundamental Limits for the Cryofixation of Water Layers

If a water layer of any thickness $h_{\mathrm{water}}$ is actively cooled from one side, the cooling rate inside the water layer is limited by the thermal diffusivity $\alpha_{\mathrm{water}}$. This imposes a fundamental limit on the cooling rate of a sample with a given size. To calculate this limit, we assume that the water is a layer that is in contact with an ideal heat sink on one side that can instantly change its temperature from room temperature ($T_{i}=20$ °C) to $T_{\infty }=-196$ °C. The boundary on the opposite side of the layer is assumed to be insulating. The layer is assumed to be unbounded in the other directions so that a one-dimensional heat conduction model can be used [40,41]. Here, using the dimensionless time $\zeta =\alpha_{\mathrm{water}}t\;h_{\mathrm{water}}^{2}$, a series approximation of the temperature *T* at position *y* in the channel can be established as follows:

(1) $$T\left(y,\zeta \right)=\left(T_{i}-T_{\infty }\right)\;\cdot \;\sum_{n=1}^{\infty }\left[A_{n}e^{-\lambda_{n}^{2}\zeta }\cos\left(\frac{\lambda_{n}y}{h_{\mathrm{water}}}\right)\right]+T_{\infty }$$

where $A_{n}$ and $\lambda_{n}$ are defined by the heat transfer of the interface and the properties of the water layer. Assuming an infinite heat transfer of the interface, this yields the following:

(2) $$\begin{array}{cc} A_{n} & =\frac{4\;\cdot \;{\left(-1\right)}^{\left(n-1\right)}}{2\pi (n-\frac{1}{2})} \end{array}$$

(3) $$\begin{array}{cc} \;\;\;\lambda_{n} & =\left(n-\frac{1}{2}\right)\;\cdot \;\pi \;n\in N \end{array}$$

**Figure 3:**
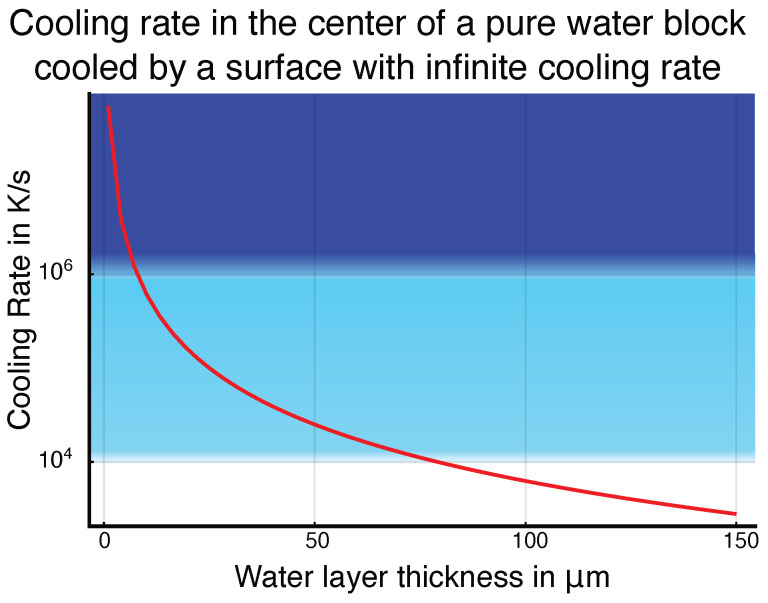
The maximum cooling rate (CR) in a sample at atmospheric pressure is limited by the thermal conduction of the water. *CR* > 10^6^ K s^−1^ of water samples cooled from one side are possible for layer heights below 7.6 μm. *CR* > 10^4^ K s^−1^ for cryofixation of biological samples is possible for thicknesses up to 50 μm. However, these values assume an infinitely high dissipation rate at the edge of the water layer, which will not be reached by any real system.

For $h_{\mathrm{water}}<50$ μm, the cooling rate in the water is well above 10^4^ K s^−1^ (see Figure 3). If a cooling rate of at least 10^6^ K s^−1^, as is necessary for the vitrification of pure water, is required, the theoretical maximum thickness is $h_{\mathrm{water}}\leq 7.6\;\mu m$. Cooling rates higher than 10^6^ K s^−1^ may theoretically be reached in regions up to 3.5μm inside a thicker water layer. Interestingly, the thickness of the water layer above does not significantly slow down the cooling rate near the boundary.

## 3. Domain Model of the Steady-State System

The vitrification of large samples in the steady-state system is only possible by using the correct materials and an optimized geometric design. Therefore, a thermal model of the system must be established. For the systems described in this paper, the dominating effect in determining the cooling rate is heat conduction. Convective heat transfer is neglected due to the small size of the microfludic system, where gravity-driven convection is insignificant (Ra≪1000) [42,43]. Also, the rapid nature of cryofixation (rates > 10^3^ K s^−1^) and the large heat transfer rate of conduction ($h_{\mathrm{conduction}}\approx 20\;W\gg h_{\mathrm{convection},\mathrm{air}}=h*A*\Delta T<1\;W$, assuming $h=30\;W\;m^{-2}\;K^{-1}$ [44], A=15 mm×5 mm and ΔT=216 K) lead to a low influence of convection in the cryofixation process.

**Figure 4:**
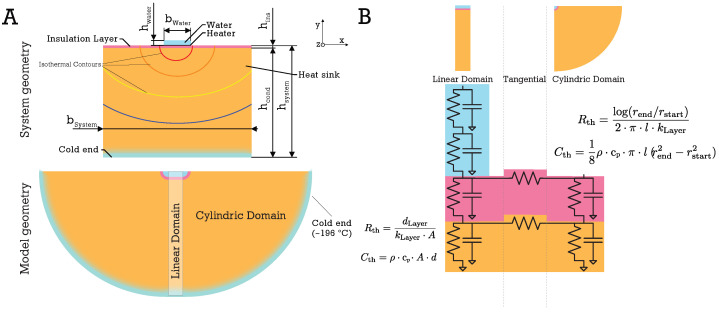
(**A**) The linear temperature distribution below the water channel and the logarithmic temperature distribution around the edge of the water channel can be modeled with a combined model represented by a network of thermal resistances and capacitances. (**B**) Each layer consists of a resistor and a capacitor for the linear domain, a resistor and a capacitor for the semi-cylindrical domain, and a resistor modeling the tangential thermal resistance between the two domains. The resistance and capacitance models for the semi-cylindrical domain are derived from the static solution of the heat conduction equation.

We model the steady-state system as a channel residing on top of a thin heater, an insulation layer, and one or multiple thermally conducting layers below, which act as a heat sink (See Figure 4A—system geometry). We base our model on the two-dimensional heat conduction equation assuming an infinite domain in the *z*-direction:

(4) $$\nabla \left(k\nabla T\right)=\rho c\frac{\partial T}{\partial t}$$

with initial and boundary conditions:

(5) $$\begin{array}{cc} T\left(y>0,t=0\right) & =T_{H}=20\;{}^{\circ}C \end{array}$$

(6) $$\begin{array}{cc} \;T\left(y=-h_{\mathrm{system}},t\right) & =T_{\mathrm{LN2}}=-196\;{}^{\circ}C \end{array}$$

To model the samples submerged in water, we assume the sample is a microfluidic channel filled with water, with the width $b_{\mathrm{water}}$ and height $h_{\mathrm{water}}$. Below the channel there is the heater layer, the insulation layer, and one or multiple heat sink layers. The dimensions of these layers are defined by the width $b_{\mathrm{system}}\gg b_{\mathrm{water}}$ and the height of the bodies below the heater $h_{\mathrm{system}}$ (see Figure 4A). As these bodies consist of different materials (insulation layer and heat sink), the thermal conductivity $k_{\mathrm{mat}}$, density $\rho_{\mathrm{mat}}$, and specific heat capacity $c_{p,\mathrm{mat}}$ of the materials are defined accordingly in their geometries. The interface of two layers is modeled as an optimal thermal contact with no contact resistance. Here, both temperature and heat flow are equal at the interface surfaces.

**Figure 5:**
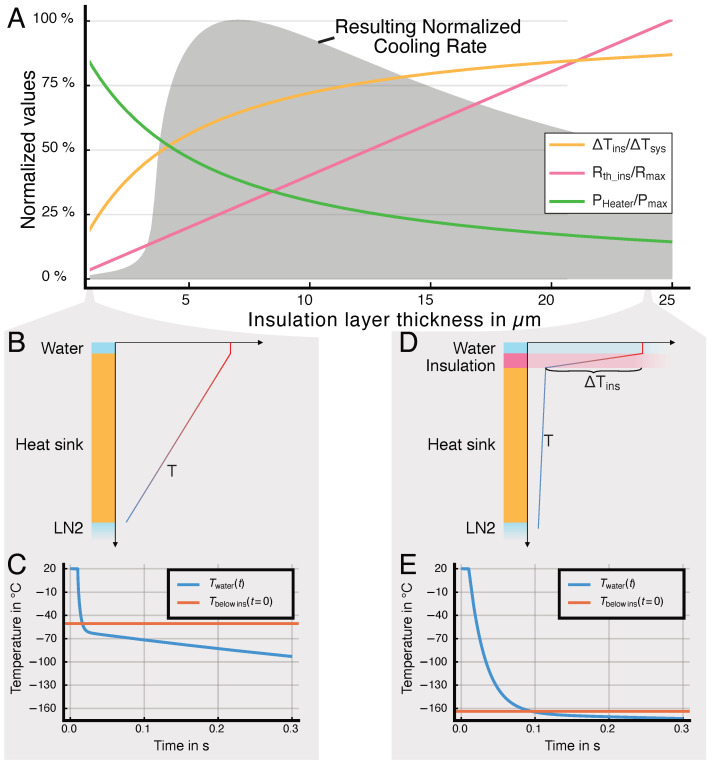
Introducing a thermal insulation layer below the heater reduces the temperature on top of the silicon layer, as the insulation layer now has a significant thermal gradient ΔT (**D**) and therefore reduces the temperature below the insulation layer $T_{\mathrm{below}\;\mathrm{ins}}$ while the heater is turned on (**A**). Accordingly, the insulation layer increases the cooling rate in the water channel, with the optimal height determined by a trade-off between temperature gradient and time constant. This is because the layers below store less energy before the cryofixation itself starts (and thus are able to conduct more energy away from the channel as soon as the heater is turned off) (**A**,**C**,**E**). The thermal resistance of the insulation layer will increase as the layer height increases. Consequently, the optimal insulation layer forms a compromise between a high temperature gradient in the insulation layer and the sufficiently short time constant $\tau =R_{\mathrm{ins}}*C_{\mathrm{ins}}$ of this layer. If there is no insulation layer, the temperature gradient is spread over the whole system, which reduces the cooling rate (**B**,**C**). The plots show the results of simulations using the domain model developed in this paper.

Since the width of the water channel is significantly smaller than that of the underlying layers, we can model the system in a cylindrical representation. Here, the water channel is defined by a cylinder with a radius equal to the real channel height. The water channel is separated from the heat sink by a thin thermal insulation layer, which will be discussed in more detail below. In the model, the insulation layer is represented by a cylindrical shell of thickness $h_{\mathrm{ins}}$ (Figure 4A). The outer surface of the heat sink, which is represented by the outermost cylindrical shell, is set constant at −196 °C. Since the system has an insulating boundary on the top and only removes heat downward, the model only incorporates half a cylinder, sectioned from 0 to π. The steady state with the heater heating may be analytically solved (see Appendix A). In this steady state, the heater does not only heat the water channel but also the layers below the heater. Consequently, the insulation layer below the heater decreases the temperature rise in the heat sink below (see Figure 5). This is critical for two reasons: first, the required heater power is reduced and second, the energy stored in the cooled layers below the insulation is reduced, improving their ability to dissipate heat once the heater is turned off.

In addition to the semi-cylindrical domain, a linear domain is introduced to model the system with wider water channels (increase in $b_{\mathrm{water}}$). Here, the water layer is no longer represented by a half-cylinder but through a rectangular domain defined by the width of the water channel $b_{\mathrm{water}}$ and its height $h_{\mathrm{water}}$. The layers below are represented by their layer thickness and the width of the water channel. Both geometries are fused to a model using a lumped-element thermal equivalent circuit in the Cauer-type configuration. Each domain of each layer is modeled by a resistor $R_{\mathrm{th}}$ and a capacitor $C_{\mathrm{th}}$ (see Figure 4B). In each layer, a tangential resistor connects the domains. We implemented this model using ngspice and automated the model preparation, simulation, and data interpretation process in Julia [45]. The model parameters, material, and computational implementation are further described in Appendix C.

A further simplification of the model is presented in Appendix B. Here, the time constants $\tau_{\mathrm{water}}$ and $\tau_{\mathrm{ins}}$ of the different layers are used to estimate the expected cooling rate using an analytical equation. This estimator is also used below to explain the influence of the heat sink material and geometry.

## 4. Device Fabrication and Assembly of the Steady-State Cryofixation System

The manufacturing process involves the fabrication of the heater on the insulation layer, the fabrication of the microfluidic channel, establishment of micro-to-macro fluidic interfaces, and the assembly of the system to ensure proper thermal contact between the channel and the heater [39,46].

The heater is fabricated on a double-sided polished silicon wafer using standard microfabrication techniques. First, the insulation layer, made of polyimide (PI-2610 from HD Microsystems), is spin-coated on the silicon wafer and cured. Next, the polyimide surface is roughened using a CF_4_ plasma to improve the adhesion of the metallization for the heater [47,48,49]. The Ti heater is then deposited by electron-beam evaporation and patterned using photolithography and wet etching. Electrical contact with the heater is established using an electron-beam-evaporated Au layer on top of the Ti heater. The backside of the heater is metallized with Cr (20 nm), Cr (100 nm), and Cu (300 nm) and soldered to the copper heat sink using indium.

In parallel to the heater fabrication, the microfluidic channel is created using a separate microfabrication process [50]. First, the negative of the microfluidic channel is patterned on a silicon wafer using SU-8 photoresist to create a master mold. A parylene coat on the master mold ensures easy demolding of the PDMS microfluidic channel. Next, the PDMS is spin-coated on the master mold, partially cured on a hotplate, and then cure-bonded to a mechanically strong PDMS structure. This enables an improved peeling process from the wafer. After peeling, the open PDMS channels are closed by plasma bonding a spin-coated 4 μm thick PDMS layer. The low thickness of this layer is critical to ensure a low thermal resistance between water and the heater. The backside of the PDMS channel structure is then plasma-bonded to a silicon chip with contact holes, which allows fluidic connection to the system.

The fabrication method offers flexibility in customizing the water channel dimensions. To modify the channel thickness, a new SU-8 master mold is created with the desired specifications. The thickness of the resulting water channel is controlled by varying the spin speed during the SU-8 deposition process. Additionally, the channel’s width and length can be tailored by altering the layout design of the master mold. This versatility allows for precise control over the channel geometry to meet specific experimental requirements.

The thermal connection between heater and channel is enabled by placing the PDMS channel on top of the heater. The channel is connected to a fluidic system that is fixed in position to ensure the stability of the thermal contact during operation. The high thermal conductivity of indium guarantees a low thermal resistance between the heater and the copper heat sink, which is placed in a reservoir that can be filled with liquid nitrogen to establish the steady state. The dimensions of the system are listed in Table 1.

## 5. Results

### 5.1. Validating the Domain Model

To validate the domain model, we compare its predictions to a geometrically accurate representation of the system in a finite element modeling simulation. Here, we compare both approaches with a set of 100 randomly generated geometries within the parameter bounds given in Table 2.

**Figure 6:**
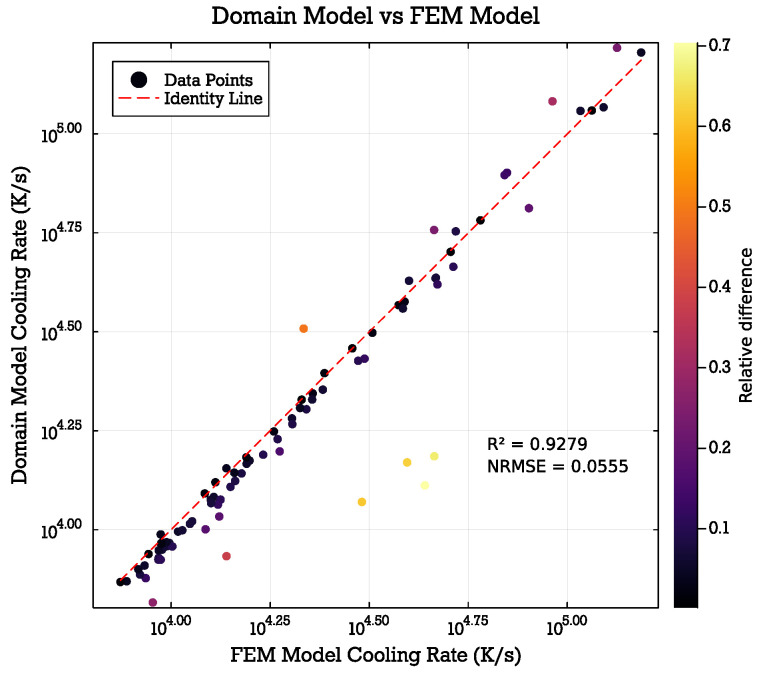
Correlation between calculated cooling rates of the domain model vs. the FEM model. An R-squared value of 0.9279 and a normalized root mean squared error (NRMSE) of 0.0555 indicate a good correlation between both models.

The domain model demonstrated strong agreement with the finite element model (FEM) results, with an R-squared value of 0.9279 (see Figure 6). This high correlation extends across the entire range of input parameters tested, suggesting robust performance under varied conditions. The close alignment between the domain model and FEM simulations indicates that the simplified approach effectively captures the key thermal conduction processes in the cryofixation system. Consequently, the domain model could serve as an efficient alternative to full FEM simulations for many applications, offering a balance between computational speed and accuracy. Several outliers can be explained by the geometry parameter combinations in these cases leading to a configuration where the given assumptions of the domain model are less valid (e.g., the assumption of a concentric temperature distribution no longer holds when the thickness of the heat sink is too small).

### 5.2. Experimental Validation of the Domain Model

In [46], Fuest et al. measured the cooling rate in a steady-state cryofixation system. Here, the cooling rate was measured by observing the change in intensity of a temperature-dependent fluorescent dye (Rhodamine B) during cooldown. The cooling rate was measured to be in the range of 2 × 10^4^ K s^−1^. To properly model the thermal conduction in the water channel, the bottom layer of the channel made of PDMS ($h_{\mathrm{pdms}}=4\;\mu m$ as described in [50]) is incorporated into the model with an equivalent electrical network layer similar to the water layer. The domain model estimates a cooling rate of 2.5 × 10^4^ K s^−1^ for this system. This is in good agreement with the measured cooling rate. Neglecting the PDMS layer in the model would result in a significantly higher predicted cooling rate, leading to a substantial deviation from the experimental observations. The remaining discrepancies between the model and the measurements can be attributed to non-ideal thermal contacts in the experimental setup. Overall, the presented model can estimate the cooling rate in the system with sufficient accuracy. Therefore, the insights from the domain model can guide the optimization of the system for a high cooling rate with a sufficiently low heater power.

**Figure 7:**
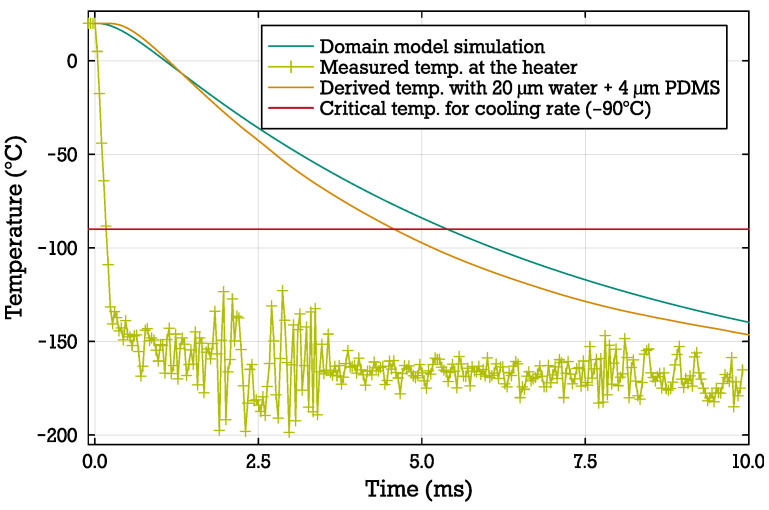
The measured temperature at the heater below the water channel is extracted by measuring the resistance during cryofixation. This temperature measurement is then used to derive the temperature at the top of the water channel by modeling the water channel as a layer structure of a 4 μm PDMS layer and a 20 μm water layer. Compared to the domain model, the cooling transient results in a comparable cooling rate in the water.

To further qualify the results, we measured the temperature change in the fabricated steady-state cryofixation system (as described in Table 1) using two experimental methods:

- **Fluorescent dye method:** Similar to the method in [46], the fluorescent intensity of the fluorescent dye Rhodamine B is used to indicate the temperature changes in the water channel. This intensity is measured using an sCMOS Camera (Andor Neo) with a frame rate of 413 Hz. A filter cube with a 546/10nm excitation filter and a 575 nm emission filter reduces the influence of ambient light and improves the signal-to-noise ratio of the fluorescence measurements. As the temperature-dependency of the intensity of the dye is only indicative for temperatures above −21 °C [51], the initial intensity change is used to indicate the cooling rate in the channel.
- **Heater resistance method:** The resistance of the actively powered heater changes with its temperature. This is possible as the resistance of the heater depends on the bulk temperature of the heater with the temperature coefficient of resistance $\alpha_{R}$. Using titanium as the heater material results in a robust heater with a significant temperature coefficient $\alpha_{R}=0.003\;\;K$ [52]. We measured both the current and voltage with 100 kHz. After an initial calibration run, the resistance is then translated to a temperature at the heater. This temperature is then used to derive the heat flow into the water channel and calculate the temperature in the channel. Here, a model of just the PDMS channel containing the water layer is used (see Figure 7).

We extract a cooling rate of 23,782 K s^−1^ from the heater resistance measurement with a channel height $h_{water}=20\;\mu m$, while the domain model yields an expected cooling rate of 20,407 K s^−1^ (see Table 3), which shows a good agreement. Optical measurements using the temperature-dependent fluorescent dye suggest a cooling rate in the range of 2 × 10^4^ K s^−1^, which is consistent with the expected cooling rate of the system.

### 5.3. Effect of the Heat Sink

The heat sink does not ideally transport the heat away from the heater. Therefore, we need to consider the impact of the solid support underneath the micro-hotplate on the cooling rate. The solid support is assumed to comprise a silicon substrate and a heat sink made of copper or other materials with high heat conductivity (e.g., diamond). Due to the finite thermal conductivity of these materials, a thermal gradient arises between the insulation layer and the LN2 reservoir.

**Figure 8:**
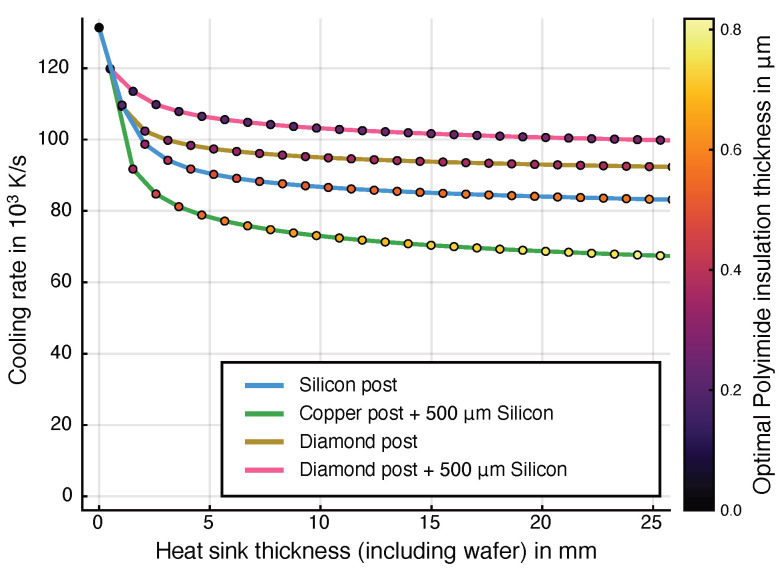
Changing the height of the heat sink below the silicon wafer only influences the achievable cooling rate in a water channel of 20 μm height significantly if the heat sink heights are comparable. A more significant effect may be seen if the material of the heat sink is changed to a material with better conduction and more optimal specific heat capacity (e.g., silicon or diamond). The simulations were performed using the domain model described in Section 3.

Both the numerical results of the domain model and the analytical representation of the cooling rate estimator (Appendix B, Equation (A23)) show that the cooling rate cannot exceed a given maximum by changing the insulation layer alone. If the time constant of the insulation layer $\tau_{\mathrm{ins}}$ is very small compared to the time constant of the water layer $\tau_{w}$ and the temperature difference in the insulation layer $\Delta T_{\mathrm{ins}}$ is sufficiently large, the dominating effect limiting the cooling rate is defined through the material properties of water. Therefore, maximizing $\Delta T_{\mathrm{ins}}$ is critical for attaining high cooling rates with the steady-state system. This is achieved by optimizing the design and the choice of materials. As the combined thickness of the silicon and the heat sink, $h_{\mathrm{heatsink}}$, only influences $\Delta T_{\mathrm{ins}}$ through a logarithmic function, its effect is very diminished compared to their effective heat conductivity $k_{\mathrm{heatsink}}$. Here, a 1000-fold value of $k_{\mathrm{heatsink}}/k_{\mathrm{ins}}$ does have a significantly stronger effect than a change in geometry. This effect can also be observed in Figure 8.

The thermal conductivity of silicon increases drastically at low temperatures. This can increase the maximum cooling rate significantly. With an optimized insulation layer, the silicon layer can reach temperatures down to the range of −180 °C to −196 °C while the heater is turned on. According to [53], the thermal conductivity of silicon reaches values of 950 W m^−1^ K^−1^ to 1670 W m^−1^ K^−1^ in this temperature range compared to 130 W m^−1^ K^−1^ at room temperature. As the heat conduction is modeled with resistors, the model incorporates this effect by replacing the resistors for the silicon layer with voltage-dependent resistors. The resulting increase in cooling rate depends on the geometry of the water channel and heater and the heat sink length. The increase ranges from 115% to 360% for silicon.

Overall, we see that the heat sink can influence the cooling rate significantly if a high thermal conductivity is provided. Changing the thickness of this layer does have a negligible influence as soon as the thickness is on a larger scale than that of the water layer.

### 5.4. Size Limitations for Cryofixation in the Steady State System

**Figure 9:**
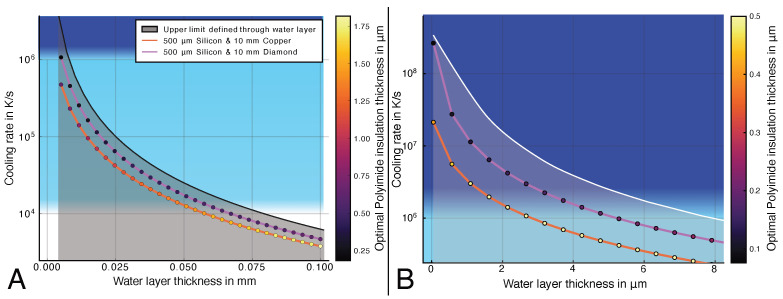
Modeled cooling rates for thick (**A**) and thin (**B**) water layer thicknesses (using the domain model) with varying heat sink materials and thicknesses. Changes in the water and sample layer thickness result in the most significant change in cooling rate in the steady-state system. The resulting curves follow the shape of the natural upper limit imposed by the cooling rate of the water layer itself, which is limited by the thermal conductivity of water.

The cooling rates in the steady-state system reflect the strong influence of the water channel height (Figure 9A). In an optimized system with a diamond heat sink, samples up to 5.4 μm would reach a cooling rate higher than 10^6^ K s^−1^. If a copper heat sink is used, this limit is reduced to 2.8 μm (Figure 9B). The insulation layer thickness needs to be optimized for each water channel configuration to reach the most optimal results. Other parameters, such as the dimensions of the geometries below the insulation layer, only have a minimal influence on the cooling rate of the system compared to the water channel thickness.

In contrast, increasing the width of the water channel $b_{\mathrm{water}}$ leads to a linear increase in necessary heater power and a decrease in the available cooling rate in the water channel. For heater and channel widths $b_{\mathrm{water}}\leq 1\;mm$ and a heat sink length of $h_{\mathrm{heatsink}}=11\;mm$, a cooling rate > 10^6^ K s^−1^ can be achieved (e.g., with $h_{\mathrm{water}}=2\;\mu m$). For larger water channel widths up to a few mm, the cooling rate stays above 10^4^ K s^−1^. The heater power is linearly proportional to the width of the heater if the insulation layer height is fixed. However, if this layer height is optimized for maximum cooling rate, the necessary heater power for wider heaters increases substantially. As all heat heater power from the heater needs to be dissipated by the LN2, the critical heat flux [54] of the liquid nitrogen can impose a power limit to the system. Therefore, it can be necessary to increase the insulation layer thickness further from the cooling rate optimum until the resulting heater power is small enough. The resulting decreased cooling rate is smaller but within the same magnitude as the system with an insulation layer height optimized for maximum cooling rate.

If the cooling rate in a cryofixation system is limited by only the thermal conductivity of the water layer, the system is able to absorb a heat flow exceeding the maximum heat flow through the water layer under the most optimal cryofixation conditions. This limiting heat flow can be estimated with the following equation:

(7) $$\dot{Q}=k_{\mathrm{water}}\;\cdot \;b_{\mathrm{water}}\;\cdot \;l_{\mathrm{water}}\;\cdot \;\frac{T_{H}-T_{N2}}{h_{\mathrm{water}}}$$

where $l_{\mathrm{water}}$ describes the length of the water channel in the *z*-direction.

In the steady-state system, the thermal resistance of the insulation layer is small enough so that this heat flow can be sustained. At the same time, this same layer guarantees a sufficiently large $\Delta T_{\mathrm{ins}}$. On the other hand, equilibrium systems dissipate this heat rapidly through transfer to a cryogenic gas or liquid. This is usually achieved by applying a cryogenic fluid directly to the sample or to the backside of the sample support. Here, the heat conductor needs to have a thermal resistance significantly lower than the thermal resistance of water to avoid impacting the cooling rate. Other established methods like plunge freezing need to not only dissipate heat from the water layer but also from a supporting structure (e.g., EM grids), which has a negative impact on achievable cooling rates.

## 6. Conclusions and Discussion

Our results demonstrate that the performance of microfluidic cryofixation systems based on solid-supported micro-hotplates can be optimized by tuning three key parameters: the insulation layer thickness ($h_{\mathrm{ins}}$), the thermal conductivity of the solid support and heat sink materials ($k_{\mathrm{heatsink}}$), and the width of the water channel and heater ($b_{\mathrm{water}}$). When these parameters are optimized, the cooling rate becomes limited only by the thermal time constants of the water layer and the insulation layer. Equilibrium systems based on cryo-spraying or plunging are mainly limited by the thickness of the sample carrier and its thermal conductivity. Here, the thermal conductivity, heat capacity, and boiling point of the cryogenic fluid (e.g., pressurized LN2) will have an increased impact compared to in microfluidic steady-state systems.

The most fundamental limit with regard to the size of the sample is dictated by the thermal conductivity of water. Including a steady-state system with limited thermal conduction further decreases the cooling rate in the sample. Using a diamond heat sink, the theoretical maximum cooling rate of an aqueous sample is diminished to 22% (for $h_{\mathrm{water}}=1\;\mu m$) and 66% (for $h_{\mathrm{water}}=50\;\mu m$). The maximum cooling rate is reduced to 13% (for $h_{\mathrm{water}}=1\;\mu m$) and 55% (for $h_{\mathrm{water}}=50\;\mu m$) for copper heat sinks. This attenuation in the cooling rate is at its maximum for low channel heights and decreases for increasing channel heights.

The heat sink can be scaled in size without significantly reducing the available cooling rate in the water layer if the water channel width is significantly smaller than the overall system size. This is possible because the insulation layer below the heater prevents substantial heating of the underlying structures while the system is in its steady state with the sample at room temperature. As the thermal resistance of the heat sink increases, the power in the steady state must be reduced by increasing the thickness of the insulation layer. The best results for large specimen sizes will be reached with an intelligent choice of the materials in this setup. As shown in Figure 9, structures up to 50 μm should be able to reach a sufficient cooling rate using a diamond–silicon–polyimide system.

Using a very wide microfluidic channel ($b_{\mathrm{water}}<1\;mm$) or reservoir on a polyimide–silicon–copper system, we expect that full vitrification of pure water samples <2 μm is possible according to our models. If larger sample sizes are desired, the copper heat sink needs to be replaced with a material of increased thermal conductivity. Similarly, increasing the width of the reservoir and, consequently, the heater will have an impact on the cooling rate in the water channel, but it will stay above 10^4^ K s^−1^ for heater widths of the same magnitude. The linear increase in heater power consumption with the heater width may be a practical limit in the steady-state system. Therefore, these more extreme configurations need an optimized system with highly conducting materials like diamond or silicon carbide.

The principle described for the steady-state system could also be interesting for other applications requiring a high cooling rate. Some examples are PCR analysis, microactuation, and micromixing as described in the introduction. Additionally, the steady-state system has the advantage of being able to create steep temperature gradients at the edge of the heater. These gradients can be used to improve the control of effects in the microfluidic domain. To actuate pico- and nano-droplets in digital microfludics, these steep gradients could increase thermocapillary effects [24,55,56,57]. Additionally, the steep temperature gradients achievable with this system could potentially enable temperature gradient focusing (TGF) techniques for separating ionic species in solution [58,59]. Furthermore, an array of micro-heaters could be used to create a pattern of locally controlled temperature zones, potentially allowing for selective deactivation of organisms in specific areas while maintaining optimal conditions for others in the same system.

## Figures and Tables

**Table 1:** Dimensions of the fabricated and assembled steady-state cryofixation system.

Parameter		Value
Water channel thickness	$h_{\mathrm{water}}$	20 μm
Water channel width	$b_{\mathrm{water}}$	100 μm
Water channel length	$l_{\mathrm{water}}$	1.8 mm
PDMS layer between heater and water	$h_{\mathrm{pdms}}$	4 μm
Insulation (PI) layer thickness	$h_{\mathrm{ins}}$	4 μm
Silicon wafer thickness	$h_{\mathrm{si}}$	500 μm
Heater width	$b_{\mathrm{heater}}$	400 μm
Heater length	$l_{\mathrm{heater}}$	3.5 mm

**Table 2:** Parameters and variation range for the validation of the domain model in comparison with the FEM model.

Parameter		Min	Max
Water layer thickness	$h_{\mathrm{water}}$	1 μm	50 μm
Insulation layer thickness	$h_{\mathrm{ins}}$	0.1 μm	15 μm
Silicon layer thickness	$h_{\mathrm{si}}$	1 μm	600 μm
Copper layer thickness	$h_{\mathrm{cu}}$	0.5 mm	15 mm
Heater and water width	$b_{\mathrm{water}}$	0.1 mm	5 mm

**Table 3:** Measured heater power and cooling rates of the cryofixation system.

	Domain Model	Measured
Heater power	10.2 W	12.0 W
Cooling rate at heater	867,009 K s^−1^	367,853 K s^−1^
Cooling rate at top of water	20,407 K s^−1^	23,782 K s^−1^

## Data Availability

Data is contained within the article.

## Appendix A. Cylindrical Static Temperature Distribution

If we assume the width of the heater and the water channel to be significantly smaller than the width of the underlying structure, we can simplify the geometry as a semi-cylindrical system. Here, we start with the heat equation in cylindrical coordinates with constant temperature in the z-direction:

(A1) $$\frac{1}{r}\frac{\partial }{\partial r}\left(rk\frac{\partial T}{\partial r}\right)=\rho c\frac{\partial T}{\partial t}$$

The water layer is represented as the innermost cylinder with $h_{\mathrm{water}}$. The subsequent layers are layered around this cylinder with their respective thicknesses.

(A2) $$\begin{array}{cc} r_{\mathrm{ins}} & =h_{\mathrm{water}}\; \end{array}$$

(A3) $$\begin{array}{cc} \;\;\;\;\;\;\;\;r_{\mathrm{si}} & =r_{\mathrm{ins}}+h_{\mathrm{ins}} \end{array}$$

(A4) $$\begin{array}{cc} \;\;\;\;r_{\mathrm{cu}} & =r_{\mathrm{si}}+h_{\mathrm{si}} \end{array}$$

(A5) $$\begin{array}{cc} r_{\mathrm{system}} & =r_{\mathrm{cu}}+h_{\mathrm{cu}} \end{array}$$

Each layer has its own temperature distribution. The boundary conditions are as follows:

(A6) $$\begin{array}{cc} T_{\mathrm{water}}\left(r\right) & =T_{\mathrm{Heater}}=20\;{}^{\circ}C \end{array}$$

(A7) $$\begin{array}{cc} \;\;\;\;\;\;\;T_{\mathrm{cu}}\left(r\right) & =T_{\mathrm{LN2}}=-196\;{}^{\circ}C \end{array}$$

Each layer interface between two layers at $r_{\mathrm{int}}$ is modeled with a perfect contact:

(A8) $$k_{1}\;\cdot \;{\left.\frac{\partial T_{1}\left(r\right)}{\partial r}\right|}_{r=r_{\mathrm{int}}}=k_{2}\;\cdot \;{\left.\frac{\partial T_{2}\left(r\right)}{\partial r}\right|}_{r=r_{\mathrm{int}}}$$

The general solution for the given PDE is as follows:

(A9) $$T_{i}\left(r\right)=\frac{C_{i_{1}}}{k_{i}}\ln\left(r\right)+C_{i_{2}}$$

Solving with the boundary conditions yields the following:

(A10) $$\begin{array}{ccc} T_{\mathrm{water}}\left(r\right) & =T_{\mathrm{heater}}\;\;\;\;\;\;\;\; & \end{array}$$

(A11) $$\begin{array}{ccc} T_{\mathrm{ins}}\left(r\right) & =\frac{T_{c1}-T_{\mathrm{heater}}}{\ln\left(\frac{r_{\mathrm{si}}}{r_{\mathrm{ins}}}\right)}\ln\left(\frac{r}{r_{\mathrm{ins}}}\right)+T_{\mathrm{heater}}\;\;\;\;\;\; & \left(r_{\mathrm{ins}}\leq r<r_{\mathrm{si}}\right) \end{array}$$

(A12) $$\begin{array}{ccc} T_{\mathrm{si}}\left(r\right) & =\frac{T_{c2}-T_{c1}}{\ln\left(\frac{r_{\mathrm{cu}}}{r_{\mathrm{si}}}\right)}\ln\left(\frac{r}{r_{\mathrm{si}}}\right)+T_{c1}\;\; & (r_{\mathrm{si}}\leq r<r_{\mathrm{cu}})\;\;\;\; \end{array}$$

(A13) $$\begin{array}{ccc} T_{\mathrm{cu}}\left(r\right) & =\frac{T_{\mathrm{LN2}}-T_{c2}}{\ln\left(\frac{r_{\mathrm{system}}}{r_{\mathrm{cu}}}\right)}\ln\left(\frac{r}{r_{\mathrm{cu}}}\right)+T_{c2}\;\; & \left(r_{\mathrm{cu}}\leq r<r_{\mathrm{system}}\right) \end{array}$$

with

(A14) $$\begin{array}{cc} T_{c1} & =-\frac{\xi_{2}T_{\mathrm{heater}}\ln\left(\frac{r_{\mathrm{cu}}}{r_{\mathrm{si}}}\right)+\xi_{1}T_{\mathrm{heater}}\ln\left(\frac{r_{\mathrm{system}}}{r_{\mathrm{cu}}}\right)+\xi_{3}T_{\mathrm{LN2}}\ln\left(\frac{r_{\mathrm{si}}}{r_{\mathrm{ins}}}\right)}{\xi_{2}\ln\left(\frac{r_{\mathrm{cu}}}{r_{\mathrm{si}}}\right)+\xi_{1}\ln\left(\frac{r_{\mathrm{system}}}{r_{\mathrm{cu}}}\right)+\xi_{3}\ln\left(\frac{r_{\mathrm{si}}}{r_{\mathrm{ins}}}\right)} \end{array}$$

(A15) $$\begin{array}{cc} T_{c2} & =-\frac{\xi_{2}T_{\mathrm{LN2}}\ln\left(\frac{r_{\mathrm{cu}}}{r_{\mathrm{si}}}\right)+\xi_{1}T_{\mathrm{heater}}\ln\left(\frac{r_{\mathrm{system}}}{r_{\mathrm{cu}}}\right)+\xi_{3}T_{\mathrm{LN2}}\ln\left(\frac{r_{\mathrm{si}}}{r_{\mathrm{ins}}}\right)}{\xi_{2}\ln\left(\frac{r_{\mathrm{cu}}}{r_{\mathrm{si}}}\right)+\xi_{1}\ln\left(\frac{r_{\mathrm{system}}}{r_{\mathrm{cu}}}\right)+\xi_{3}\ln\left(\frac{r_{\mathrm{si}}}{r_{\mathrm{ins}}}\right)} \end{array}$$

(A16) $$\xi_{1}=k_{\mathrm{ins}}k_{\mathrm{si}}\;\xi_{2}=k_{\mathrm{cu}}k_{\mathrm{ins}}\;\xi_{1}=k_{\mathrm{cu}}k_{\mathrm{si}}$$

Using this equation system, the temperature distribution during heater operation can be modeled as cylindrical. To make the system more compliant with the actual setup, we only take the top half section of the cylinder, which does not influence the temperature distribution but the calculated power:

(A17) $$P=-k_{\mathrm{si}}\pi L\frac{T_{c2}-T_{c2}}{\ln\left(\frac{r_{\mathrm{cu}}}{r_{\mathrm{si}}}\right)}$$

This system can also be used to establish the equivalent resistances and capacitances for a Cauer-type network:

(A18) $$R_{th,si}=\frac{\Delta T}{P}=\frac{\ln\left(\frac{r_{\mathrm{cu}}}{r_{\mathrm{si}}}\right)}{k_{\mathrm{si}}\pi L}$$

(A19) $$C_{th,si}=\frac{Q_{stored}}{\Delta T}=c_{p}m=\frac{1}{2}c_{p}\rho \pi L\left(r_{cu}^{2}-r_{si}^{2}\right)$$

## Appendix B. Limiting Case: Estimator for a Narrow Microchannel on a Micro-Hotplate with an Ideal Heat Sink

In the case of a narrow microfluidic channel and an ideal heat sink, a simple circuit model provides some interesting insights. Therefore, in this section we consider a model that includes only the water layer and the insulation layer. From the static cylindrical model (see Appendix A) we know that the temperature difference in the insulation layer can be expressed as

(A20) $$\Delta T_{\mathrm{ins}}=\frac{\frac{k_{\mathrm{heatsink}}}{k_{\mathrm{ins}}}\ln\left(\frac{h_{\mathrm{ins}}\;+\;h_{w}}{h_{w}}\right)}{\ln\left(\frac{h_{\mathrm{heatsink}}\;+\;h_{\mathrm{ins}}\;+\;h_{w}}{h_{\mathrm{ins}}\;+\;h_{w}}\right)+\frac{k_{\mathrm{heatsink}}}{k_{\mathrm{ins}}}\ln\left(\frac{h_{\mathrm{ins}}\;+\;h_{w}}{h_{w}}\right)}\;\cdot \;\left(T_{H}-T_{\mathrm{LN2}}\right)$$

where $T_{H}=20\;{}^{\circ}C$ is the temperature of the heater in the steady state and $T_{\mathrm{LN2}}=-196\;{}^{\circ}C$ is the temperature of the LN2. Using this definition, we reduce the network to the following formulation: We model the water layer ($R_{w}$,$C_{w}$) and the insulation layer ($R_{\mathrm{ins}}$,$C_{\mathrm{ins}}$) both as a single RC network using the Cauer representation. The heater between both layers is represented as a power source $P_{h}\left(t\right)$ and the temperature below the insulation layer is set to $T_{H}-\Delta T_{\mathrm{ins}}$. We can now express the system using the transfer function of the water temperature $T_{w}$ and the heater power $P_{h}$ in the complex frequency domain as follows:

(A21) $$\begin{array}{c} \frac{T_{w}}{P_{h}}\left(s\right)=\frac{R_{\mathrm{ins}}}{1+\left(R_{\mathrm{ins}}C_{\mathrm{ins}}+R_{w}C_{w}+R_{\mathrm{ins}}C_{w}\right)s+R_{\mathrm{ins}}R_{w}C_{\mathrm{ins}}C_{w}s^{2}}=\frac{R_{\mathrm{ins}}}{1+\xi_{1}s+\xi_{2}s^{2}} \end{array}$$

As the layers described in this system are very thin, $\xi_{1}$ is dominant compared to $\xi_{2}$. Omitting $\xi_{2}$ and renaming $\xi_{1}=\tau_{\mathrm{sys}}$ as a time constant yields the transient temperature of the water layer after an inverse transform:

(A22) $$T_{w}\left(t\right)=\Delta T_{\mathrm{ins}}*e^{-\frac{t}{\tau_{\mathrm{sys}}}}-\Delta T_{\mathrm{ins}}+T_{H}$$

The used time constant $\tau_{\mathrm{sys}}$ is the sum of the time constants of both layers $\tau_{\mathrm{ins}}=R_{\mathrm{ins}}C_{\mathrm{ins}}$ and $\tau_{w}=R_{w}C_{w}$ and a combined time constant $\tau_{c}=R_{\mathrm{ins}}C_{w}$ that describes the time it takes for the stored heat (as described by $C_{w}$) to flow through the resistance of the insulation layer $R_{\mathrm{ins}}$. This third time constant becomes relevant if the insulation layer height increases and slows down the heat flow through the insulation layer.

These time constants now form the estimator for the cooling rate. At $t=\tau_{1}$, 63% of the temperature difference $\Delta T_{\mathrm{ins}}$ is surpassed. Therefore, we extract the cooling rate with

(A23) $$CR\approx \frac{0.63\;\cdot \;\Delta T_{\mathrm{ins}}}{\tau_{\mathrm{sys}}}=\frac{0.63\;\cdot \;\Delta T_{\mathrm{ins}}}{\tau_{\mathrm{ins}}+\tau_{w}+\tau_{c}}$$

## Appendix C. Model Parameters and Computational Methods

The domain model allows the following parameters to be set before computation:

**Table A1:** Computational input parameters for the domain model.

Parameter	Symbol	Unit
System width (x-direction)	$b_{\mathrm{system}}$	m
System length (z-direction)	$L_{\mathrm{system}}$	m
**For each layer (Water, PDMS, insulation, heat sink):**		
Layer thickness	$h_{\mathrm{water}}$	m
Thermal conductivity of water	$k_{\mathrm{water}}$	W m^^−1^^ K^−1^
Specific heat capacity of water	$c_{p,\mathrm{water}}$	J kg^−1^ K^−1^
Density of water	$\rho_{\mathrm{water}}$	kg m^−3^

**Table A2:** Thermal material parameters used in the simulations with the domain model and the reference FEM model [60,61,62,63,64].

	Water	PDMS	Polyimide
Thermal conductivity	0.560 W m^^−1^^ K^−1^	0.15 W m^^−1^^ K^−1^	0.14 W m^^−1^^ K^−1^
Density	999.9 kg m^−3^	970 kg m^−3^	700 kg m^−3^
Specific heat capacity	4219.9 J kg^−1^ K^−1^	1460 J kg^−1^ K^−1^	2329 J kg^−1^ K^−1^
	Silicon	Copper	Diamond
Thermal conductivity	130 W m^^−1^1^ K^−1^	401 W m^^−1^1^ K^−1^	2900 W m^^−1^1^ K^−1^
Density	2329 kg m^−3^	8960 kg m^−3^	3500 kg m^−3^
Specific heat capacity	700 J kg^−1^ K^−1^	384 J kg^−1^ K^−1^	509 J kg^−1^ K^−1^

The domain model was implemented using ngspice (version 34) for circuit simulation. Model preparation, simulation, and data interpretation were automated using Julia (version 1.6.3) [45]. The parameters were converted into resistances and capacitors using the given formulae of the Cauer model and then inserted into the netlist for the ngspice simulation. Temperature-dependent thermal conductivites are represented as voltage-dependent functions that are based on an approximated analytic function of the thermal conductivity dependent on the temperature.

Finite element modeling (FEM) simulations for model validation were performed using COMSOL Multiphysics (version 6.0). The Heat Transfer in Solids interface was used for both a static and a time-dependent study.
